# Integrated Multi‐Omics Analysis Identifies PDK4 and ACOT1 as Metabolic Hub Genes Associated With Myocardial Fibrosis in Diabetic Cardiomyopathy

**DOI:** 10.1155/jdr/1952672

**Published:** 2026-07-30

**Authors:** Huan Liu, Guanming Qi, Shengrong Ouyang, Feifei Ma

**Affiliations:** ^1^ Department of Biochemistry and Immunology, Capital Center for Children′s Health, Capital Medical University, Capital Institute of Pediatrics, Beijing, China, shouer.com.cn; ^2^ Internal Medicine, Guthrie Lourdes Hospital, Binghamton, New York, USA

**Keywords:** ACOT1, cardiac fibroblasts, diabetic cardiomyopathy, metabolic reprogramming, myocardial fibrosis, PDK4

## Abstract

Diabetic cardiomyopathy (DCM) is a critical pathological driver of heart failure in diabetic patients, primarily characterized by progressive myocardial fibrosis. Nevertheless, the core molecular network linking upstream metabolic dysregulation to the aberrant activation of downstream cardiac fibroblasts remains largely elusive. In this study, we combined in vivo and in vitro approaches with bioinformatics analysis. A high‐fat diet‐induced mouse model of diabetic myocardial fibrosis was established, and transcriptome sequencing was performed to screen for hub genes, which were subsequently validated in two independent DCM datasets. Single‐cell RNA sequencing revealed that PDK4 and ACOT1 were upregulated in cardiac fibroblasts under pathological conditions. In vitro experiments confirmed that high glucose induced the expression of PDK4, ACOT1, and fibrotic markers in human primary cardiac fibroblasts. Molecular docking predicted a potential interaction between PDK4 and ACOT1. Collectively, our findings identify PDK4 and ACOT1 as evolutionarily conserved metabolic hub genes associated with myocardial fibrosis in DCM, suggesting a putative “metabolism–fibrosis axis” and providing potential therapeutic targets.

## 1. Introduction

Diabetes mellitus (DM) has become a global public health challenge, with cardiovascular complications remaining as the leading cause of disability and mortality among diabetic patients [1–3]. Diabetic cardiomyopathy (DCM), a myocardial dysfunction independent of coronary artery disease and hypertension, has gained increasing recognition as a critical complication [4, 5]. Epidemiological data indicate that the risk of heart failure in diabetic patients is 2 to 5 times higher than in nondiabetic individuals, with DCM contributing substantially to this excess risk of these cases [6]. The pathological progression of DCM is complex, evolving from early metabolic disturbances and diastolic dysfunction to late‐stage systolic failure and overt heart failure [7, 8]. Central to this process is myocardial fibrosis, characterized by excessive collagen deposition and aberrant remodeling of the cardiac extracellular matrix. This fibrotic remodeling increased myocardial stiffness, impaired diastolic relaxation, and ultimately drives the progression toward heart failure [9, 10].

The pathogenesis of myocardial fibrosis involves a complex interplay among multiple cell types and signaling pathways [11]. In the metabolic context of hyperglycemia, hyperlipidemia, and insulin resistance, the heart is subjected to multiple insults, including lipotoxicity, endoplasmic reticulum stress, and chronic low‐grade inflammation, which collectively drive fibrotic remodeling [12, 13]. Although traditional views emphasize cardiomyocyte injury as central to fibrosis, recent studies increasingly highlight the pivotal role of cardiac fibroblasts as the “executors” of fibrosis [14, 15]. Nonetheless, the specific molecular bridges linking upstream metabolic disturbances to the downstream fibrotic activation program, particularly the core hub gene network governing fibrosis progression, remain to be fully elucidated.

To elucidate the molecular network driving myocardial fibrosis in DCM, the integration of high‐throughput sequencing and bioinformatics approaches enables genome‐wide interrogation of critical regulators implicated in disease progression. Nevertheless, findings derived from a single model are often limited in generalizability. Hence, validation using independent external datasets is therefore essential to enhance the reliability of the discoveries and their potential for clinical translation.

Based on this rationale, this study is aimed at integrating animal models, cellular experiments, and public transcriptomic datasets through a multi‐omics analysis strategy to systematically identify and validate hub genes that play a central role in diabetic myocardial fibrosis. This work is expected to provide new insights into the molecular mechanisms underlying diabetic myocardial fibrosis and to lay a theoretical foundation for the future development of targeted antifibrotic therapies.

## 2. Materials and Methods

### 2.1. Animal Model Establishment and Evaluation

The animal study protocol received review and approval from the Animal Ethics Committee of the Capital Institute of Pediatrics (Approval No. KSDWLL2017033). Eight‐week‐old male C57BL/6J mice, obtained from GemPharmatech Co. Ltd., were randomly allocated into two dietary groups: a normal diet (ND) group (10% fat‐derived energy, *n* = 3) and a high‐fat diet (HFD) group (60% fat‐derived energy, *n* = 4). The HFD (D12492, Research Diets, United States) was used to induce a model that recapitulates key features of human Type 2 diabetes, including insulin resistance and lipotoxicity. Body weight was monitored and recorded at 2‐week intervals throughout the experimental period.

After 12 weeks of feeding, an oral glucose tolerance test (OGTT) was performed on all mice. Following a 6‐h fast, glucose solution (2 g/kg) was administered intraperitoneally, and blood glucose levels were measured via tail vein blood sampling at 0, 30, 60, and 120 min postinjection.

At the experimental endpoint, left ventricular myocardial tissue was collected. One portion of the tissue was fixed immediately in 4% paraformaldehyde, embedded in paraffin, sectioned, and stained using a Masson′s trichrome staining kit (Solarbio, China) to evaluate interstitial collagen deposition (blue areas). Stained sections were observed under an upright light microscope, and the fibrosis area was semiquantitatively analyzed using Image J software. The remaining myocardial tissue was rapidly frozen in liquid nitrogen and then transferred to a −80°C freezer for subsequent RNA extraction.

### 2.2. Transcriptome Sequencing and Bioinformatics Analysis

Total RNA was isolated from myocardial tissues of ND and HFD mice using SparkZol reagent (SparkJade, China). Qualified samples were subsequently subjected to transcriptome sequencing (RNA‐seq) on an Illumina NovaSeq6000 platform at Shanghai Biotechnology Corporation (Shanghai, China). Raw RNA‐seq reads were quality‐filtered using Trimmomatic (Version 0.39) and aligned to the mouse reference genome (mm10) using HISAT2 (Version 2.2.1). Gene‐level read counts were quantified using featureCounts (Version 2.0.1). Normalization was performed using the trimmed mean of M‐values (TMM) method implemented in the “edgeR” package. Differential expression analysis was performed using the “limma” package with voom transformation. Batch effects were not applicable as all samples were processed in a single sequencing run. The criteria for differential expression were |log₂FC| ≥ 1 and *p* value < 0.05; no formal multiple‐testing correction was applied for the initial screening, but all key candidate genes were validated in independent datasets.

### 2.3. GO and KEGG Enrichment Analysis

Gene Ontology (GO) functional enrichment and Kyoto Encyclopedia of Genes and Genomes (KEGG) pathway enrichment analyses were performed for the identified differentially expressed genes (DEGs) using the bioinformatics online platform (https://www.bioinformatics.com.cn/). Terms with a *p* value < 0.05 were considered significantly enriched.

### 2.4. Protein–Protein Interaction (PPI) Network Construction and Hub Gene Identification

The DEGs were imported into the STRING database (https://cn.string-db.org/) to construct a PPI network, with a minimum interaction confidence score set at > 0.15. The resulting network file was then imported into Cytoscape software (Version 3.10.1). Using the MCC (Maximal Clique Centrality) algorithm within the cytoHubba plugin, the centrality of each node was calculated. The top 5 genes ranked by centrality were selected as candidate hub genes.

### 2.5. External Dataset Validation Analysis

Two DM datasets (rat and mice), GSE5606 [16] (Control = 7, DM = 7) and GSE210611 [17] (Control = 3, DM = 3), were downloaded from the Gene Expression Omnibus (GEO) database (https://www.ncbi.nlm.nih.govgeo/). Differential expression analysis was performed on each dataset separately using the “limma” package in R, applying the same filtering criteria as used for the internal data (|log₂(fold change)| ≥ 1 and *p* value < 0.05). A Venn diagram was generated to identify the DEGs common to both datasets. Subsequently, the aforementioned PPI network construction and cytoHubba analysis pipeline were repeated on these common DEGs to identify the top 5 hub genes, which were then compared with the results derived from the HFD mouse model.

### 2.6. Temporal Expression and Cell‐Type–Specific Analysis of Hub Genes

The gene expression dataset GSE4745 [18] (Control = 4, DM = 4), which includes myocardial tissues from different time points during diabetes progression, was downloaded. Expression data of the hub genes identified in this study were extracted, and a clustered heat map was generated using the “pheatmap” R package to visualize their expression patterns in control and diabetic groups at Days 3, 28, and 42. Temporal expression curves for each hub gene were also plotted. Furthermore, the single‐cell RNA sequencing dataset GSE213337 [19] (Control = 1, DM = 1) for diabetic myocardial fibrosis was downloaded. Standard preprocessing, dimensional reduction, clustering, and cell‐type annotation were performed using the “Seurat” R package. Cell types were annotated using canonical marker genes as follows: fibroblasts (Vim, Pdgfra, Col1a1), cardiomyocytes (Tnnt2, Myh6), endothelial cells (Pecam1, Cdh5), smooth muscle cells (Acta2, Tagln), mural cells (Rgs5, Notch3), endocardial cells (Npr3, Gja5), neutrophils (S100a8, Ly6g), macrophages (Cd68, Adgre1), T/B cells (Cd3e, Cd79a), glial cells (Gfap, Sox10), and proliferating cells (Mki67, Top2a). We focused on the fibroblast population and evaluated the expression of the five hub genes.

### 2.7. In Vitro Cellular Experimental Validation

Primary human cardiac fibroblasts (Catalog #6340, ScienCell, United States) were maintained in complete fibroblast medium supplemented with 10% fetal bovine serum. To mimic the diabetic milieu, cells were randomly allocated to three treatment groups: normal glucose (NG, 5.5 mM glucose), high glucose (HG, 33 mM glucose), and an osmotic control (MA, 5.5 mM glucose plus 27.5 mM mannitol), with the latter maintaining total osmolarity equivalent to the HG group. Cells were exposed to these conditions for 5 days.

### 2.8. Real‐Time Quantitative Polymerase Chain Reaction (RT‐qPCR)

RNA extraction and real‐time quantification of total RNA were performed by isolating RNA from cells using SparkZol reagent (AC0101) and subsequently reverse‐transcribing it into complementary DNA (cDNA). With *β* actin serving as the internal reference gene, RT‐qPCR was performed using SYBR Green premix reagent on a QuantStudio real‐time PCR system. The mRNA targets examined included PDK4, ACOT1, HMGCS2, NR1D1, CYP1A1, TGF‐*β*1, and COL3A1. The relative gene expression levels were calculated using the 2^−^ *ΔΔ*Ct method. The primer sequences used are detailed in Table 1.

**Table 1:** qPCR primer sequences.

	Forward	Reverse
PDK4	CGGCTTGCCAATTTCTCGTC	GCCAGGTTCTTTGGTTCCCT
ACOT1	GAGCTGGAGGTGCTGGATG	AAAGGGCCCAGGTTCTGGC
HMGCS2	CATCAACTCCCTGTGCCTGA	TCCATCCAGTTGGCAGCATT
NR1D1	TGCGGACCCTGAACAACAT	GGGGAGGGAGGCAGGTATTTA
CYP1A1	AATTTCGGGGAGGTGGTTGG	AGGCATTCAGGGAAGGGTTG
TGF‐*β*1	ACCTGCCACAGATCCCCTAT	CTCCCGGCAAAAGGTAGGAG
Collagen III	CGCCCTCCTAATGGTCAAGG	TTCTGAGGACCAGTAGGGCA
*β* actin	CTCGCCTTTGCCGATCC	ATCCTTCTGACCCATGCCC

### 2.9. Western Blot

Western blot analysis was performed with the following primary antibodies: anti‐PDK4 (Immunoway, cat# YN5701, rabbit, 1:1000), anti‐ACOT1 (Immunoway, cat# YT0085, rabbit, 1:1000), anti‐TGF *β*1 (Immunoway, cat# YM8257, rabbit, 1:1000), anti‐Vimentin (Cell Signaling Technology, cat# 5741, rabbit, 1:1000), anti‐Collagen III (Immunoway, cat# YM8111, rabbit, 1:1000), and anti‐*β* actin (Cell Signaling Technology, cat# 4967, rabbit, 1:1000) as the loading control. All primary antibodies were validated by the manufacturers for specificity via knockout/knockdown validation or peptide competition assays. Each western blot experiment was performed with three independent biological replicates. Band intensity was quantified using ImageJ software (Version 1.53t) and normalized to the corresponding *β* actin band intensity for each sample.

### 2.10. Molecular Docking

The interaction between PDK4 and ACOT1 was simulated and analyzed by molecular docking in this study. The procedure was as follows: The three‐dimensional structures of PDK4 (ID: Q16654) and ACOT1 (ID: Q86TX2) were obtained from the UniProt database (https://www.uniprot.org/). Rigid docking simulation was performed using the HDOCK online server (http://hdock.phys.hust.edu.cn/), with PDK4 as the ligand and ACOT1 as the receptor. Amino acid residues within a binding distance of 5 Å in the docking conformation were extracted to analyze potential interaction sites. The docking complex structure was preprocessed and visualized using PyMOL software to illustrate the key residues at the PPI interface.

### 2.11. Statistical Analysis

**Figure 1:**
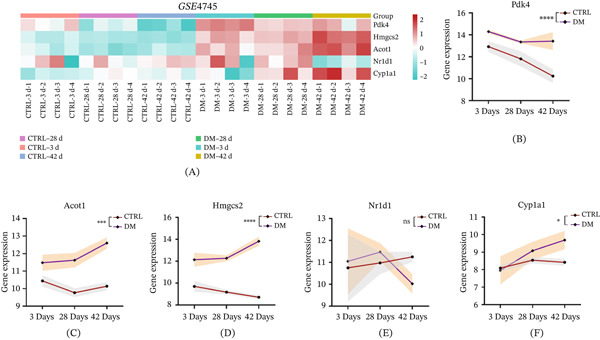
Temporal expression dynamics of candidate hub genes in a longitudinal diabetic cardiomyopathy dataset. (A) Clustered heatmap showing the expression profiles of five hub genes (Pdk4, Hmgcs2, Acot1, Nr1d1, Cyp1a1) in control and diabetic groups at days 3, 28, and 42. (B‐F) Line graphs depicting the temporal expression patterns of individual hub genes (B: Pdk4, C: Acot1, D: Hmgcs2, E: Nr1d1, F: Cyp1a1) across the three time points. Data are presented as mean ± SEM.  ^∗^ *p* < 0.05,  ^∗∗^ *p* < 0.01,  ^∗∗∗^ *p* < 0.001,  ^∗∗∗∗^ *p* < 0.0001.

All experimental data are presented as the mean ± standard error of the mean (SEM). Comparisons between two groups were performed using an unpaired two‐tailed Student′s *t*‐test. For the temporal expression analysis in Figure 1, because multiple time points were compared, we adopted a conservative interpretation and only highlighted genes showing consistent trends across all time points; the robustness of the findings was further supported by validation in other datasets. For comparisons among multiple groups (NG, HG, and MA), one‐way analysis of variance (ANOVA) was used, followed by Tukey′s post hoc test for between‐group differences. All statistical analyses were performed using GraphPad Prism software (Version 9.0). No formal power calculation was performed for sample size determination due to the exploratory nature of this pilot study; sample sizes were chosen based on previously published studies in the field. A *p* value < 0.05 was considered statistically significant.

## 3. Results

### 3.1. Establishment and Characterization of a HFD‐Induced Obese Mouse Model of Diabetic Myocardial Fibrosis

**Figure 2:**
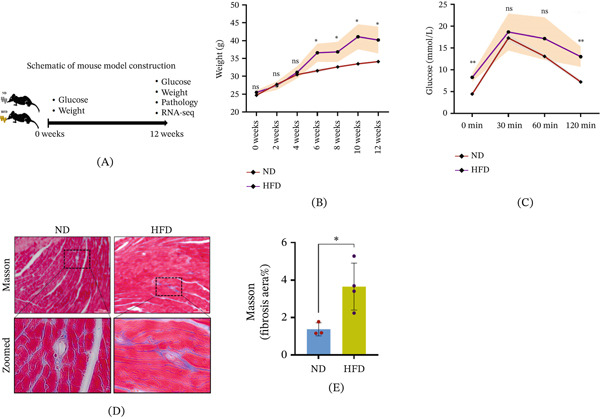
Establishment and phenotypic characterization of the high‐fat diet‐induced diabetic myocardial fibrosis mouse model. (A) Schematic diagram of the experimental timeline. (B) Body weight progression of mice in the ND and HFD groups over 12 weeks (ND = 3, HFD = 4). (C) Oral glucose tolerance test (OGTT) at week 12. (D) Representative images of Masson′s trichrome staining of left ventricular myocardial sections from ND and HFD mice. Collagen deposition is shown in blue. Scale bar = 100 *μ*m. (E) Quantitative analysis of myocardial fibrosis area from (D). Data are presented as mean ± SEM,  ^∗^ *p* < 0.05,  ^∗∗^ *p* < 0.01.

To investigate diabetic myocardial fibrosis, a mouse model was established by feeding C57BL/6 mice either a ND or a HFD for 12 weeks (Figure 2A). Weekly body weight monitoring revealed a significant and sustained increase in the body weight of HFD‐fed mice compared to the ND group throughout the study period (Figure 2B). At the end of the 12th week, an OGTT showed that blood glucose levels were significantly higher in HFD mice at both the fasting (0 min) and 120‐min time points compared to ND controls. Although the differences at intermediate time points (30 and 60 min) did not reach statistical significance, blood glucose levels in the HFD group remained consistently elevated (Figure 2C). Histological analysis of left ventricular myocardial tissue via Masson′s trichrome staining confirmed a significant increase in myocardial fibrosis in HFD mice compared to ND mice (Figure 2D,E).

### 3.2. Transcriptomic Analysis Reveals Differential Gene Expression Profiles in Myocardium of Obese Diabetic Mice

**Figure 3:**
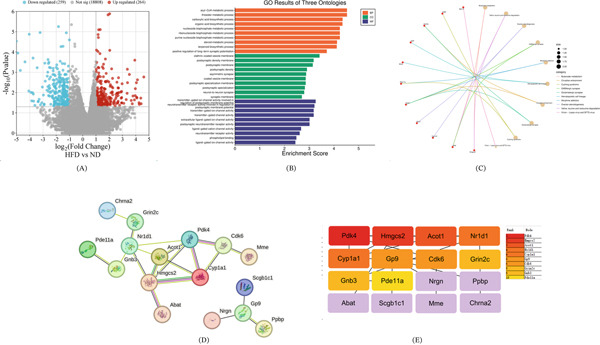
Transcriptomic profiling identifies key genes and pathways in diabetic cardiomyopathy. (A) Volcano plot of differentially expressed genes (DEGs) in HFD vs. ND mouse hearts. (B) GO enrichment analysis of DEGs across Biological Process, Cellular Component, and Molecular Function ontologies. (C) KEGG pathway enrichment analysis of DEGs presented as a bubble plot. (D) Protein‐protein interaction network of DEGs constructed using the STRING database. (E) Identification of the top 5 hub genes (Pdk4, Hmgcs2, Acot1, Nr1d1, and Cyp1a1) from the PPI network.

To investigate the underlying mechanisms, RNA sequencing was performed on myocardial tissues from ND and HFD mice. Differential expression analysis (criteria: |log₂FC| ≥ 1, *p* value < 0.05) revealed a distinct transcriptomic signature in the hearts of HFD mice (Figure 3A). GO enrichment analysis demonstrated significant enrichment of the DEGs across all three ontologies: Biological Process, Cellular Component, and Molecular Function (Figure 3B). Specifically, these genes were enriched in lipid metabolism‐related biological processes, neuron‐associated cellular components such as the postsynaptic membrane, and molecular functions like neurotransmitter receptor activity, suggesting substantial metabolic reprogramming and potential aberrant neuro‐like receptor signaling in the cardiac tissue of the model. KEGG pathway enrichment analysis further indicated that the differential genes were significantly clustered in key pathways including “Valine, leucine and isoleucine degradation,” “Glutamatergic synapse,” and “Morphine addiction” (Figure 3C). A PPI network constructed using the STRING database revealed potential interactions among the differential genes (Figure 3D). Subsequent network centrality analysis using the cytoHubba plugin in Cytoscape identified the top 5 hub genes: Pdk4, Hmgcs2, Acot1, Nr1d1, and Cyp1a1 (Figure 3E).

### 3.3. Identification and Validation of Hub Genes in External DCM Datasets

**Figure 4:**
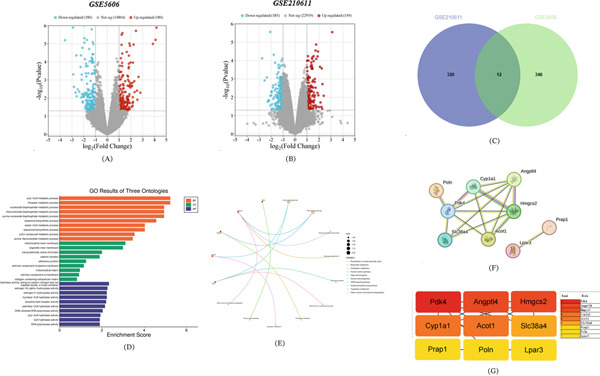
Identification and validation of hub genes in external diabetic cardiomyopathy datasets. (A, B) Volcano plots showing differentially expressed genes (DEGs) in the (A) GSE5606 and (B) GSE210611 datasets. Significantly up‐ and downregulated genes (|log^2^ *F* *C*| ≥ 1, *p* < 0.05) are highlighted in red and blue, respectively. (C) Venn diagram illustrating the overlap of DEGs between the two external datasets, identifying 12 common DEGs. (D, E) Functional enrichment analysis of the 12 common DEGs. (D) Bar graph of the top enriched Gene Ontology terms across Biological Process, Cellular Component, and Molecular Function. (E) Bubble plot of enriched KEGG pathways; bubble size and color represent gene count and significance, respectively. (F) Protein–protein interaction network of the 12 common DEGs constructed using the STRING database. (G) The top 5 hub genes (Pdk4, Angptl4, Hmgcs2, Cyp1a1, and Acot1) identified from the PPI network using the cytoHubba plugin.

To validate our findings, we analyzed two independent DCM gene expression datasets (GSE5606 and GSE210611). Differential expression analysis was performed on each dataset separately (criteria: |log^2^ *F* *C*| ≥ 1, *p* value < 0.05), with results presented as volcano plots (Figure 4A,B). Venn diagram analysis identified 12 DEGs common to both datasets (Figure 4C). GO enrichment analysis of these common DEGs revealed significant enrichment in terms such as “acetyl‐CoA metabolic process,” “mitochondrial inner membrane,” and “acyl‐CoA hydrolase activity” (Figure 4D). KEGG pathway analysis further demonstrated that these genes were significantly clustered in pathways related to metabolism and cardiac remodeling, including “Biosynthesis of unsaturated fatty acids,” “Butanoate metabolism,” “Cholesterol metabolism,” and the “PPAR signaling pathway” (Figure 4E). A PPI network was constructed using the STRING database (Figure 4F), and the top 5 hub genes—Pdk4, Angptl4, Hmgcs2, Cyp1a1, and Acot1—were identified using the cytoHubba plugin in Cytoscape (Figure 4G). Notably, four of these genes (Pdk4, Hmgcs2, Acot1, and Cyp1a1) overlapped with the hub genes identified in our HFD mouse model, indicating a high degree of consistency in their pivotal roles across different models and species.

### 3.4. Temporal Expression Patterns Reveal Dynamic Changes of Hub Genes During DCM Progression

To analyze the longitudinal expression dynamics of the five hub genes, we extracted their expression profiles from the GSE4745 dataset. In addition to the targeted hub gene analysis, we performed a comprehensive differential expression analysis of the entire GSE4745 dataset (criteria: |log₂FC| ≥ 1, *p* < 0.05) and confirmed that the overall transcriptomic landscape was consistent with published analyses of this dataset. Focusing on our five hub genes, clustered heat map visually displays their overall expression profiles at Days 3, 28, and 42 postdiabetes induction (Figure 1A). Further analysis via individual temporal expression curves revealed that compared to the control group, Pdk4, Acot1, and Hmgcs2 exhibited consistent and significant upregulation in the diabetic group at all time points examined (Figure 1B–D). In contrast, the expression patterns of Nr1d1 and Cyp1a1 displayed a distinct dynamic: Their expression levels were more variable and showed an intermingled pattern across time points and between groups, without a clear and consistent trend of regulation (Figure 1E,F).

### 3.5. Expression of Hub Genes in Cardiac Fibroblasts During Diabetic Myocardial Fibrosis

**Figure 5:**
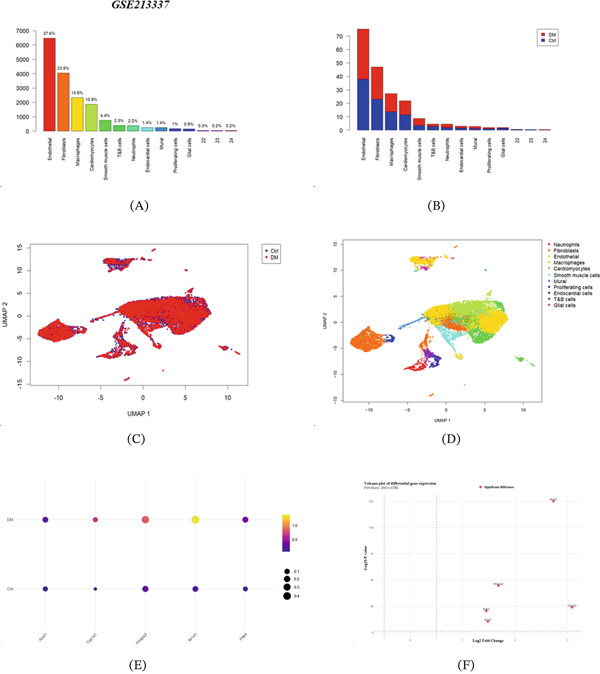
Single‐cell RNA sequencing analysis of hub gene expression in cardiac fibroblasts. (A) Bar chart showing the proportion of all cell types in the GSE213337 dataset. (B) Bar chart of cellular composition colored by annotated cell types. (C) UMAP plot showing the distribution of cells from CTRL and DM groups. (D) UMAP visualization highlighting the distribution of cardiac fibroblasts in CTRL versus DM samples. (E) Bubble plot visualizing the expression of the five hub genes in fibroblasts from control and disease groups. Dot size represents the percentage of expressing cells, and color intensity indicates the average expression level. (F) Volcano plot displaying the differential expression of the five hub genes in fibroblasts between disease and control states. Statistical significance is indicated.

To delineate the expression features of hub genes at single‐cell resolution, we analyzed a single‐cell RNA sequencing dataset (GSE213337) of diabetic myocardial fibrosis. Initial cellular composition analysis revealed that cardiac fibroblasts constituted a significant proportion in both groups (Figure 5A), and their distribution across samples was clarified following cell type annotation (Figure 5B). UMAP visualization clearly demonstrated the distribution differences in the overall transcriptomic landscape between the control and diabetic groups (Figure 5C), as well as the specific distribution pattern of cardiac fibroblasts between the two conditions (Figure 5D). Focusing on the fibroblast population, we further evaluated the expression of five hub genes (Pdk4, Acot1, Hmgcs2, Nr1d1, and Cyp1a1). A bubble plot visually indicated that, compared to the control group, these genes exhibited higher average expression levels and a greater percentage of expressing cells in the disease‐state fibroblasts (Figure 5E). A volcano plot further confirmed that several of these hub genes were significantly upregulated in cardiac fibroblasts during diabetic myocardial fibrosis (Figure 5F). These results provide direct single‐cell‐level evidence that key hub genes are expressed in cardiac fibroblasts—the central effector cells in DCM—offering cellular‐level support for their functional role in driving the fibrotic process. While these data demonstrate that hub genes including PDK4 and ACOT1 are expressed in cardiac fibroblasts under disease conditions, definitive demonstration of cell‐type specificity relative to other cardiac cell types (cardiomyocytes, endothelial cells, and immune cells) requires further validation through immunostaining or FACS‐sorted population analysis.

### 3.6. In Vitro Functional Validation in Human Primary Cardiac Fibroblasts

**Figure 6:**
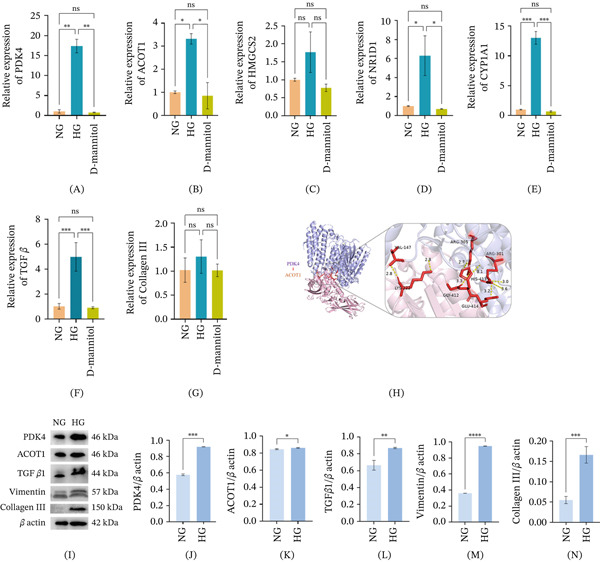
High glucose induces the expression of hub genes and fibrotic markers in human primary cardiac fibroblasts in vitro. (A–G) mRNA expression levels of the indicated genes measured by qRT‐PCR in cells treated as described. (H) Molecular docking model illustrating the predicted interaction interface between PDK4 and ACOT1. (I) Representative western blot images showing protein levels of PDK4, ACOT1, TGF‐*β*1, Vimentin, Collagen III, and *β*‐actin under different treatment conditions. (J–N) Quantification of the protein expression levels normalized to *β*‐actin from (I). Data are presented as mean ± SEM of *n* = 3 independent experiments.  ^∗^ *p* < 0.05,  ^∗∗^ *p* < 0.01,  ^∗∗∗^ *p* < 0.001,  ^∗∗∗∗^ *p* < 0.0001.

To validate the function of hub genes at the cellular level, we established an in vitro model using human primary cardiac fibroblasts. Cells were cultured for 5 days under normal glucose (NG, 5.5 mM), high glucose (HG, 33 mM), or an osmotic control condition (MA, 33 mM mannitol). RT‐qPCR results demonstrated that high glucose stimulation significantly induced the mRNA expression of hub genes PDK4, ACOT1, NR1D1, CYP1A1, as well as the classical fibrotic marker TGF β1 (Figure 6A–E, F). In contrast, isosmotic control treatment did not elicit similar changes, indicating a glucose‐specific effect. Although the increases in HMGCS2 and COL3A1 mRNA levels did not reach statistical significance, a consistent upward trend was observed (Figure 6E,G). Given that the prior PPI network suggested a strong potential interaction between PDK4 and ACOT1, we performed molecular docking simulation, which visually suggested a potential PPI interface between them, providing a hypothesis‐generating basis for future experimental validation Figure 6H. Furthermore, we validated these findings at the protein level by western blot. Compared to the NG condition, the protein expression levels of PDK4, ACOT1, TGF *β*1, Vimentin, and Collagen III were all significantly increased in cardiac fibroblasts exposed to the high glucose environment (Figure 6I–N). These results, obtained in a functional cellular model, confirm that high glucose can specifically activate hub genes including PDK4 and ACOT1, as well as fibrotic pathways, providing supporting evidence for their pathogenic role in diabetic myocardial fibrosis.

## 4. Discussion

DCM is a leading cause of heart failure and mortality in diabetic patients, characterized by progressive myocardial fibrosis [20]. This study integrated animal models, multi‐omics bioinformatics analysis, and in vitro cellular experiments to systematically explore the key molecular network driving diabetic myocardial fibrosis. We identified and validated a set of hub genes, with PDK4 and ACOT1 at the core, which are activated in the heart, particularly in cardiac fibroblasts, under hyperglycemic metabolic stress. These genes are closely associated with lipid metabolic reprogramming and may interact to jointly regulate the downstream fibrotic process. This discussion will elaborate on the potential mechanisms, scientific significance, and clinical translational value of these core findings.

Our HFD mouse model successfully recapitulated the core features of Type 2 diabetes, including weight gain, impaired glucose tolerance, and significant myocardial interstitial fibrosis, a phenotype highly consistent with clinical DCM progression [21]. Subsequent transcriptomic analysis revealed widespread gene expression dysregulation in the myocardial tissue of HFD mice. Interestingly, GO and KEGG enrichment analyses were not limited to traditional pathways of myocardial hypertrophy or fibrosis but were significantly enriched in pathways related to energy metabolism and neuronal signaling, such as “Valine, leucine and isoleucine degradation” and “Glutamatergic synapse.” This suggests that in early‐stage diabetes, the heart undergoes profound systemic metabolic substrate utilization and local signaling microenvironment remodeling in response to lipotoxicity and hyperglycemic stress. This metabolic reprogramming may be a precursor to subsequent structural remodeling, such as fibrosis [22, 23]. In this context, the hub genes identified through PPI network analysis (Pdk4, Hmgcs2, Acot1, Nr1d1, and Cyp1a1) are mostly directly involved in fatty acid oxidation, ketone body generation, and circadian rhythm regulation, further confirming the central role of metabolic disorders in driving DCM pathology. For instance, PDK4 promotes a shift from glucose oxidation to fatty acid oxidation in cardiomyocytes by inhibiting the pyruvate dehydrogenase complex, a switch that may exacerbate lipid accumulation and oxidative stress under insulin‐resistant conditions [24, 25].

Findings from a single model are often limited by species specificity and modeling methods. To enhance the reliability and generalizability of our discoveries, we validated them in two independent DCM gene expression datasets (GSE5606 and GSE210611). Convincingly, the top 5 hub genes (PDK4, ANGPTL4, HMGCS2, CYP1A1, and ACOT1) reselected from the 12 common DEGs showed a high degree of overlap (4/5) with the mouse model results. This consistency across species (mouse/rat) and models (diet induced/drug induced) strongly suggests that genes like PDK4 and ACOT1 play an evolutionarily conserved and crucial role in the fibrotic progression of DCM. Functional analysis of the common DEGs revealed significant enrichment in “acetyl‐CoA metabolic process,” “PPAR signaling pathway,” and “biosynthesis of unsaturated fatty acids.” The PPAR signaling pathway is central to regulating cellular lipid metabolism, inflammation, and fibrosis, whereas acetyl‐CoA is a key metabolite connecting glucose, lipid, and amino acid metabolism [26]. Together, this paints a clear picture: Diabetic metabolic disturbances perturb core metabolic regulatory pathways like PPAR, alter cardiac acetyl‐CoA homeostasis and lipid metabolic flux, and subsequently activate a hub gene network represented by PDK4 and ACOT1.

Traditionally, DCM research has focused on metabolic dysfunction in cardiomyocytes [27, 28]. However, the direct executors of fibrosis are activated cardiac fibroblasts [29–31]. Our temporal expression analysis (GSE4745) showed that PDK4, ACOT1, and HMGCS2 exhibited stable and significant upregulation at various time points after diabetes induction (Days 3, 28, and 42), whereas the expression patterns of Nr1d1 and Cyp1a1 were more variable. This suggests that the former three are more stable drivers consistently involved in DCM pathogenesis. More importantly, single‐cell RNA sequencing analysis (GSE213337), localized the high expression of hub genes like PDK4 and ACOT1 to cardiac fibroblast subpopulations under disease conditions at cellular resolution. This finding has paradigm‐shifting significance, extending the functional context of metabolic hub genes (PDK4/ACOT1) from cardiomyocytes to cardiac fibroblasts. We hypothesize that the diabetic environment (hyperglycemia and hyperlipidemia) also imposes strong metabolic stress on cardiac fibroblasts. These cells may undergo metabolic adaptation by upregulating genes like PDK4, and this adaptation process may unexpectedly drive their transformation into a profibrotic myofibroblast phenotype. This provides a novel cellular perspective for understanding how “metabolic signals directly instruct the fibrotic program.”

To test the above hypothesis, we conducted functional validation in human primary cardiac fibroblasts. High‐glucose stimulation specifically (not hyperosmotic) upregulated the mRNA and protein levels of genes such as PDK4, ACOT1, and TGF *β*1, accompanied by increased expression of Vimentin and Collagen III. This directly proves that high glucose can drive cardiac fibroblasts toward an activated state synthesizing extracellular matrix (ECM) by activating metabolic genes like PDK4 and ACOT1. Particularly noteworthy, molecular docking simulations predicted a potential PPI interface between PDK4 and ACOT1. PDK4 is responsible for metabolic switching, whereas ACOT1 (acyl‐CoA thioesterase 1) hydrolyzes long‐chain fatty acyl‐CoAs, potentially regulating lipid signaling molecules or alleviating lipotoxicity. Their computationally predicted interaction raises the hypothesis that they may form a functionally coupled “metabolic sensing‐regulation module” within cardiac fibroblasts. This prediction, however, requires experimental validation through co‐immunoprecipitation (Co‐IP), fluorescence resonance energy transfer (FRET), or surface plasmon resonance (SPR) studies in future work.

This study also have limitations. We acknowledge that the initial RNA‐seq discovery phase used a modest sample size (ND = 3, HFD = 4) and that no formal multiple‐testing correction was applied for the DEG screening. This may introduce potential false positives. However, this limitation is substantially mitigated by the multitiered validation strategy, which included two independent external datasets, cross‐species consistency, single‐cell resolution verification, and functional in vitro experiments. All key genes, especially PDK4 and ACOT1, were consistently identified across multiple platforms and models, providing strong support for the reliability of our conclusions. First, the HFD mouse model primarily reflects insulin resistance and lipotoxicity, insufficiently modeling the pathological features of late‐stage hyperglycemia. Second, while strongly suggestive, this study has not yet conducted causal gain/loss‐of‐function experiments (e.g., gene knockout or overexpression) in animal or cellular models to definitively prove the functional importance of PDK4/ACOT1, which is a crucial next step. Third, the predicted interaction between PDK4 and ACOT1 requires further experimental confirmation, such as Co‐IP or FRET.

We acknowledge that the present study is primarily correlative and does not establish causation. While our multi‐omics and in vitro data strongly associate PDK4 and ACOT1 with fibrotic activation, definitive proof of their causal role requires loss‐of‐function and gain‐of‐function experiments. Future studies should employ CRISPR‐Cas9‐mediated knockout or adenoviral overexpression of PDK4 and ACOT1 in cardiac fibroblasts, followed by assessment of fibrotic markers (TGF *β*1, Collagen I/III, and *α*‐SMA) and functional assays (collagen gel contraction and migration) to establish causality. Additionally, fibroblast‐specific conditional knockout mice would be valuable to evaluate the in vivo functional significance of these genes in diabetic myocardial fibrosis.

While our single‐cell RNA‐seq analysis localized PDK4 and ACOT1 expression to cardiac fibroblast subpopulations, we acknowledge that this does not conclusively establish cell‐type specificity. Future studies should employ immunofluorescence co‐staining of PDK4/ACOT1 with fibroblast markers (e.g., Vimentin and PDGFR*α*) or FACS‐based sorting of cardiac fibroblasts followed by RT‐qPCR to rigorously compare expression levels across different cardiac cell types.

Our findings strongly align with recent frontiers in DCM research. For instance, studies increasingly emphasize the metabolic plasticity of cardiac fibroblasts and their role in fibrosis, as well as the concept of “metabolic memory” [32, 33]. The core genes identified here, PDK4 and NR1D1, are both key components of the circadian clock, suggesting that circadian rhythm disruption may be another potential axis linking systemic diabetic metabolic abnormalities to local cardiac fibrosis, consistent with recent research on circadian regulation of cardiac metabolism and fibrosis [34, 35].

Based on the above discussion, we propose a working hypothesis of a “metabolism–fibrosis axis”: Systemic diabetic metabolic disturbances alter metabolic substrate utilization in the heart (particularly in cardiac fibroblasts), activating pathways like PPAR and inducing the expression of hub genes such as PDK4 and ACOT1. These genes interact, driving metabolic reprogramming and activation of cardiac fibroblasts, enhancing their response to classic fibrotic signals like TGF *β*, and ultimately leading to pathological ECM deposition. We acknowledge, however, that this model remains speculative. Alternative interpretations are equally plausible: The observed metabolic changes could be a consequence of the fibrotic process rather than its cause, or both metabolic and fibrotic alterations could be parallel downstream consequences of hyperglycemia without a direct causal link between them. Distinguishing among these possibilities requires future mechanistic studies, including temporal perturbation experiments and pathway‐specific interventions. Future research should delve into the downstream effectors of the PDK4/ACOT1 module and evaluate their therapeutic potential as novel targets for “precision antifibrosis” that inhibit fibrosis without compromising normal metabolic function.

Our findings align with the emerging paradigm of multi‐omics–driven precision medicine in cardiovascular disease. Recent work has proposed rigorous tiered pipelines for translating proteomic and genomic discoveries into cardiovascular therapeutics, emphasizing the importance of cross‐platform validation and functional prioritization [36]. In this context, our integrative approach—combining transcriptomic discovery across multiple models and species, single‐cell resolution analysis, and in vitro functional validation—represents a practical application of such a multitiered validation framework. The identification of PDK4 and ACOT1 as conserved metabolic hub genes in DCM illustrates how systematic multi‐omics integration can prioritize novel targets for subsequent mechanistic and therapeutic investigation, consistent with the broader vision of precision cardiology.

## 5. Conclusion

The multi‐omics investigation identifies PDK4 and ACOT1 as evolutionarily conserved metabolic hub genes critically associated with myocardial fibrosis in DCM. Cross‐species bioinformatics validation and single‐cell transcriptomic analysis reveal their specific upregulation in cardiac fibroblasts under diabetic stress. In vitro functional validation confirms that hyperglycemia selectively induces the expression of PDK4, ACOT1, and key fibrotic markers in human cardiac fibroblasts. Molecular docking computationally predicts a potential interaction between these proteins, which warrants future experimental validation. Our findings support a hypothetical “metabolism–fibrosis axis” paradigm wherein metabolic stress activates the PDK4/ACOT1 module within cardiac fibroblasts, translating systemic diabetic perturbations into a profibrotic transcriptional program. This hypothesis, while supported by correlative evidence from multiple independent datasets and in vitro models, requires definitive testing through causal gain and loss of function experiments. This study shifts the focus of metabolic dysregulation in DCM to the fibroblast compartment, revealing PDK4 and ACOT1 as promising therapeutic targets for precise antifibrotic intervention.

## Author Contributions

Conceptualization: Feifei Ma, Huan Liu, Shengrong Ouyang, and Guanming Qi; data curation: Huan Liu and Guanming Qi; formal analysis: Huan Liu and Feifei Ma; funding acquisition: Shengrong Ouyang and Feifei Ma; investigation: Huan Liu and Guanming Qi; methodology: Huan Liu, Guanming Qi, and Feifei Ma; project administration: Shengrong Ouyang and Feifei Ma; resources: Shengrong Ouyang and Feifei Ma; software: Huan Liu and Guanming Qi; supervision: Shengrong Ouyang and Feifei Ma; validation: Huan Liu and Feifei Ma; visualization: Huan Liu; writing – original draft: Huan Liu; writing – review and editing: Shengrong Ouyang and Feifei Ma. Huan Liu and FeiFei Ma contributed equally to this work.

## Funding

The study was supported by the Capital Institute of Pediatrics Foundation (FX‐2019‐04).

## Disclosure

All authors have read and approved the final version of the manuscript. Feifei Ma and Shengrong Ouyang, as co‐corresponding authors, had full access to all of the data in this study and take complete responsibility for the integrity of the data and the accuracy of the data analysis.

## Ethics Statement

All animal experiments were reviewed and approved by the Animal Ethics Committee of the Capital Institute of Pediatrics (Approval No. KSDWLL2017033) and were carried out in compliance with relevant ethical guidelines. All procedures were performed in accordance with the ARRIVE guidelines. No human subjects were involved in this study; therefore, informed consent was not applicable.

## Conflicts of Interest

The authors declare no conflicts of interest.

## Data Availability

The datasets generated and analyzed during this study are not publicly available due to ongoing research and intellectual property considerations but are available from the corresponding author on reasonable request.
